# The Italian Catquest-9SF cataract questionnaire: translation, validation and application

**DOI:** 10.1186/s40662-016-0043-9

**Published:** 2016-04-28

**Authors:** Eirini Skiadaresi, Giuseppe Ravalico, Silvio Polizzi, Mats Lundström, Miguel González-Andrades, Colm McAlinden

**Affiliations:** University Eye Clinic of Trieste, Ospedale Maggiore, Trieste, Italy; Department of Ophthalmology, Singleton Hospital, ABM University Health Board, Swansea, SA2 8QA UK; Department of Clinical Sciences, Ophthalmology, Faculty of Medicine, Lund University, Lund, Sweden; Cornea & Refractive Surgery Service, Massachusetts Eye & Ear Infirmary, Department of Ophthalmology, Harvard Medical School, Boston, USA; ABM University Health Board, Swansea, UK; Flinders University, Bedford Park, Adelaide, South Australia Australia; Wenzhou Medical University, Wenzhou, Zhejiang China

**Keywords:** Catquest-9SF, Questionnaires, Patient-reported outcomes, Cataract, Cataract surgery, Rasch analysis

## Abstract

**Background:**

To validate the Catquest-9SF questionnaire in Italian, assess the change in visual disability with cataract surgery and determine the correlation between pre-operative Catquest-9SF scores and Lens Opacities Classification System (LOCS) III cataract grading.

**Methods:**

Prospective, questionnaire validation study. The Catquest-9SF questionnaire was forward and back translated and completed by 209 Italian patients before and three months following cataract surgery. Rasch analysis was used to assess its psychometric properties.

**Results:**

The Italian Catquest-9SF demonstrated ordered response categories, unidimensionality (item fit statistics range: 0.73–1.34), adequate person separation (2.04), and no differential item functioning. Mistargeting was evident with a mean difference in item difficulty and person ability of 2.04 logits but improved with inclusion of pre-operative data only. There was a statistically significant (Friedman tests, *p* < 0.001) median improvement in visual disability of 1.92, 3.57, 1.44 and 2.94 logits in patients undergoing first eye surgery with and without ocular comorbidity, and second eye surgery with and without ocular comorbidity respectively. There was no statistically significant difference in the improvements among the four groups (Kruskal-Wallis H test, *X*^*2*^*(3)* = 5.445, *p* = 0.142). There was no correlation between Catquest-9SF scores and nuclear opalescence (*r*_s_ = 0.049, *p* = 0.478), nuclear colour (*r*_s_ = 0.008, *p* = 0.909), cortical (*r*_s_ = 0.066, *p* = 0.341), and posterior subcapsular components (*r*_s_ = 0.048, *p* = 0.494).

**Conclusions:**

The Italian Catquest-9SF demonstrated good psychometric properties and is suitable for use in Italian speaking patients. There were similar improvements in visual disability in patients undergoing first or second eye surgery, with or without ocular comorbidity. There was no correlation between pre-operative Catquest-9SF scores and LOCS III cataract grading.

**Précis:**

The Catquest-9SF cataract questionnaire was translated and validated into Italian. It demonstrates robust psychometric properties and is available for use with Italian speaking patients.

## Background

Cataract surgery is a commonly performed operation with high levels of efficacy and low complication rates [1–3]. Akin to many surgical procedures, it attracts a high level of interest in terms of research and innovation, such as intraocular lens development and femtosecond laser technology [4–7]. Determining the success of the operation is of the utmost importance. Whilst objective measures such as visual acuity and residual refractive error are important outcomes from the surgeon’s perspective, the ability to perform day-to-day tasks is one of the most important outcomes from the patient’s perspective. It is important to note that some patients, who have had uneventful cataract surgery with gains in visual acuity, may still not be satisfied [8]. This may be due to a variety of complex reasons such as patient expectations and doctor-patient communication [9]. To that end, there have been many questionnaires developed to measure various latent traits following cataract surgery. Many have also been refined via the rigors of Rasch analysis; the gold standard technique in questionnaire validation [10]. The Catquest-9SF questionnaire is one such example.

The Catquest-9SF measures visual disability on an interval scale and may be used in patients with cataract or following cataract surgery. It contains nine questions (items); two global items and seven difficulty items. [11, 12] It demonstrates robust psychometric properties and has been shown to be the most responsive to cataract surgery in comparison to 15 other Rasch-refined cataract questionnaires [13]. It has also been selected as the instrument of choice in the European Registry of Quality Outcomes for Cataract and Refractive Surgery (EUREQUO) database for the assessment of cataract outcomes. This is a project aiming to improve treatment, standards of care and to develop evidence-based guidelines for cataract and refractive surgery across Europe [14]. The questionnaire is available in Swedish, English, German and Chinese, and it is currently being translated into a number of other languages.

The purpose of this study is to translate and culturally adapt the Catquest-9SF cataract questionnaire into Italian and to subsequently assess its psychometric properties via Rasch analysis. An additional aim is to assess the change in visual disability in patients undergoing cataract surgery in an Italian public hospital. This will be assessed in four patient groups: patients undergoing first and second eye surgery and those with and without ocular comorbidities. The final aim is to compare pre-operative Catquest-9SF questionnaire scores (visual disability) with the cataract severity grading.

## Methods

### Translation process

There were five members in the translation team: one translation coordinator, two professional native English speaking translators, one professional native Italian speaking translator and one native Italian ophthalmologist fluent in English. The Catquest-9SF was initially translated from English to Italian by the professional native Italian speaking translator and the native Italian ophthalmologist in an independent manner creating two translated versions (forward translation). Subsequently, the two translators met with the translation coordinator to compare the two versions and to produce a reconciled version. This reconciled Italian version was then translated from Italian back to English by the two professional native English speaking translators (back translation) in an independent manner. The five members of the translation team then met to review the forward and back translated versions and to discuss any discrepancies. A final Italian translated version of the Catquest-9SF was produced. Further, a group of subjects in the target population were asked to review the questionnaire to ensure adequate understanding.

### Subjects

Following translation of the Catquest-9SF into Italian, patients undergoing cataract surgery at the University Eye Clinic of Trieste in Italy were invited to participate in this questionnaire validation study. Patients completed the questionnaire before surgery and at three months following surgery. Questionnaire completion was via interview with an ophthalmology resident. The post-surgery time period was chosen to ensure patients had adequate time to acquire new residual refractive error correction in the form of spectacles and to adapt to their new visual situation. This therefore should enable the patients to make a more accurate self-reported assessment of their visual disability or lack of it following surgery. To avoid statistical bias, data was collected for only one eye of each patient even if they required cataract surgery in both eyes. However, patients who required cataract surgery in both eyes did not have second eye surgery within the three month study period. The study was approved by the University of Trieste Medical Ethics Committee, the tenets of the Declaration of Helsinki were followed and patients signed a consent form.

Exclusion criteria consisted of difficulty with the Italian language or comprehension, vulnerable patient groups and patients less than 18 years of age. Patients with ocular comorbidities were included and were defined as any potentially visually significant eye disease. All patients had a complete ophthalmic examination before and after surgery and the following data were collected: age, gender, uncorrected distance visual acuity (UDVA), spherical equivalent (SE) refraction, spectacle corrected distance visual acuity (CDVA), Quality of Vision (QoV) questionnaire [15, 16] scores and Lens Opacities Classification System (LOCS) III grading (decimal scale) of the cataract. The Zeiss IOLMaster 500 (Carl Zeiss Meditec AG, Jena, Germany) biometer was used to determine the optimal dioptric power of intraocular lens and the target refraction was zero in all patients. Surgery was by phacoemulsification with monofocal intraocular lens implantation.

### Statistical analyses

Rasch analysis is a psychometric model now widely used in the development of new questionnaires and in the assessment of existing questionnaires [17, 18]. Rasch analysis converts ordinal rating scale observations into linear measures and it links how patients interact statistically with the use of probability measurement models [19–21]. A polytomous (multiple options) Andrich rating scale model (ln[*p*(*x*)/*p*(*x* - *1*)]) using joint maximum likelihood estimation was applied using commercial software (Winsteps, version 3.70.0.2, Chicago, IL, USA). The Catquest-9SF consists of two rating scales. The Wolfe and Chiu stacking procedure was employed whereby pre-operative and post-operative data were stacked together for analysis.

Rasch analysis was used to assess the Italian Catquest-9SF questionnaire for response category ordering, item fit statistics, principal components analysis, precision, differential item functioning and targeting. Response category ordering was assessed graphically with category probability curves by observing if the category calibration increased in an orderly fashion. All nine items of the Catquest-9SF have four response options that signify three thresholds. Item fit statistics (infit and outfit) enable an assessment of questionnaire unidimensionality i.e., does the questionnaire measure one construct, in this case visual disability or is it measuring more than one construct. It is fundamental that questionnaires (or subscales) measure only one construct. An ideal fit would be a residual mean square of 1.0 but a suggested acceptable range for clinical purposes is 0.50–1.50 [22]. Unidimensionality can be further complemented by a principal components analysis of the residuals and a cutoff of 2.0 eigenvalue units was used in this study. Questionnaire precision was assessed via the person separation ratio, which indicates the ability of the questionnaire to differentiate along its scale. A greater person separation indicates greater questionnaire precision and a minimum acceptable person separation ratio is 2.0. The questionnaire item calibration should be comparable across different patient groups. These differences between different groups of patients are known as differential item functioning. This was assessed by comparing first and second eye cataract surgery, and in addition by comparing with and without ocular comorbidity. Significant differential item functioning was classified as values greater than 1.0 logits. Finally, targeting of the questions to the patient sample was assessed to establish if the questions are appropriate for patients with cataract and following cataract surgery. This can be assessed graphically by observing the spread along the person-item map and by the difference in the mean question and patient scores. A mean difference in magnitude of 1 logit indicates significant mistargeting [23].

The Shapiro-Wilk test was used to assess data for normality. Friedman tests were used to compare the statistical significance of changes in visual disability measures pre-operatively to post-operatively. Kruskal-Wallis H tests were used to determine if the change in visual disability from pre- to post-operative was statistically significant between the four groups. Spearman rank correlation coefficients were calculated to assess the correlation between pre-operative Catquest-9SF scores and LOCS III cataract grading [24]. Initially, a direct comparison for each component of the LOCS III grading to the Catquest-9SF scores was calculated. To reduce the limitations of this approach, patients were then grouped into their individual cataract morphologies: nuclear, cortical or posterior subcapsular. The cutoff for nuclear and cortical cataract was 2.5 and a cutoff of 0.1 was used for posterior subcapsular cataracts. This is in line with previous studies [25]. Mixed cataracts were excluded from the analysis. A very strong correlation was classified as a coefficient >0.8, moderately strong within the range 0.5–0.8, fair within the range 0.3–0.5, and poor <0.3. Statistics were calculated using SPSS (IBM SPSS Corporation, version 20, Somers, NY, USA) and Excel (Excel 2010, Microsoft, Redmond, WA, USA). A *P*-value of less than 0.05 was considered statistically significant.

## Results

Two hundred and twelve patients were recruited. Three patients had complicated surgery (posterior capsular tear and anterior vitrectomy) and were excluded from the subsequent analysis; hence the data from 209 eyes were included in the analysis. The mean (± standard deviation) age was 74.2 (±8.7) years and there were 87 males and 122 females. There were 113 right eyes and 96 left eyes. 106 eyes were first eye surgeries and 103 eyes were second eye surgeries. Fifty-three eyes had ocular comorbidities and 156 eyes had no ocular comorbidities. Detailed information with regards to the pre- and post-operative (where applicable) UDVA, SE refraction, CDVA, QoV scores, LOCS III cataract grading including comparisons between the four patient groups (first and second eye surgery, with and without ocular comorbidities) are available in a previous publication involving the same cohort of patients [26]. Patient demographics and clinical information are displayed in Table 1.

**Table 1:** Demographic data and Lens Opacities Classification System (LOCS) III cataract grading

	First eye (*n* = 106)		Second eye (*n* = 103)	
	Without comorbidity (*n* = 78)	With comorbidity (*n* = 28)	Without comorbidity (*n* = 78)	With comorbidity (*n* = 25)
Male/Female^a^	33/45	15/13	30/48	9/16
Age (mean ± SD)^b^ (years)	72.6 (±8.9)	75.0 (±8.6)	75.2 (±9.0)	75.3 (±6.7)
Median nuclear opalescence (range)	4.15 (4.00)	4.95 (5.79)	4.75 (4.90)	4.55 (4.10)
Median nuclear colour (range)	4.05 (4.50)	4.50 (4.50)	4.55 (3.60)	5.00 (4.30)
Median cortical (range)	2.70 (4.00)	2.70 (5.40)	2.85 (4.20)	2.60 (4.00)
Median posterior subcapsular (range)	0.10 (4.80)	1.55 (5.80)	0.10 (4.40)	0.75 (4.60)

*Abbreviations*: *SD* = standard deviation; *ANOVA* = analysis of variance

^a^Females and males were not equally distributed across groups (*P* = 0.004, Chi-square test)

^b^Mean age was comparable across questionnaires (*P* > 0.05 [ANOVA])

### Psychometric assessment

**Fig. 1:**
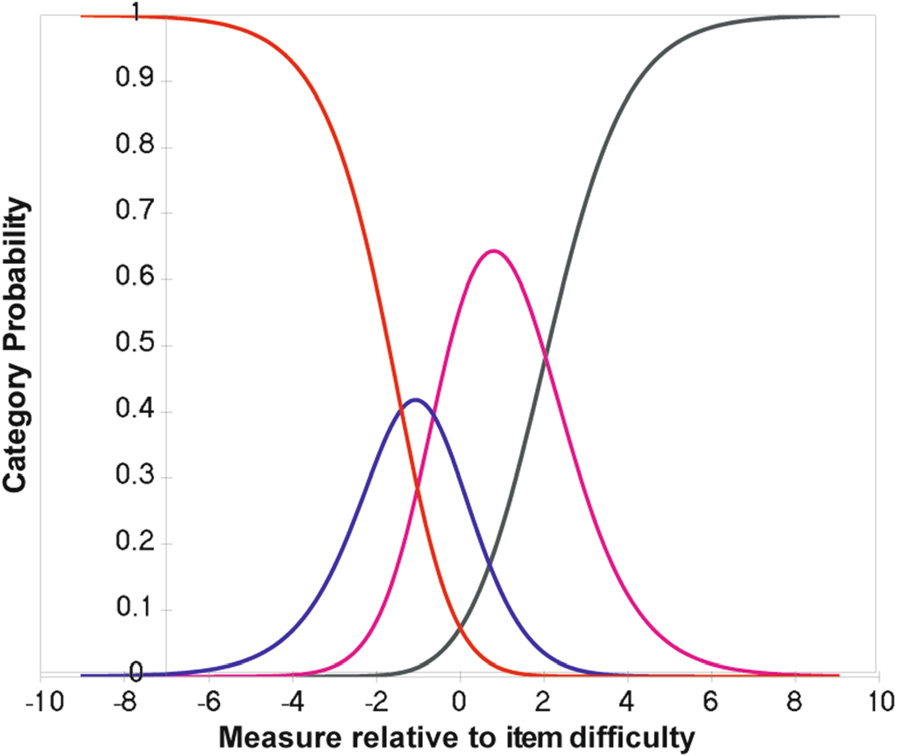
First rating scale category probability curve. This figure displays the category probability curves for the first rating scale which includes items 1 and 3–9, demonstrating ordered thresholds

**Fig. 2:**
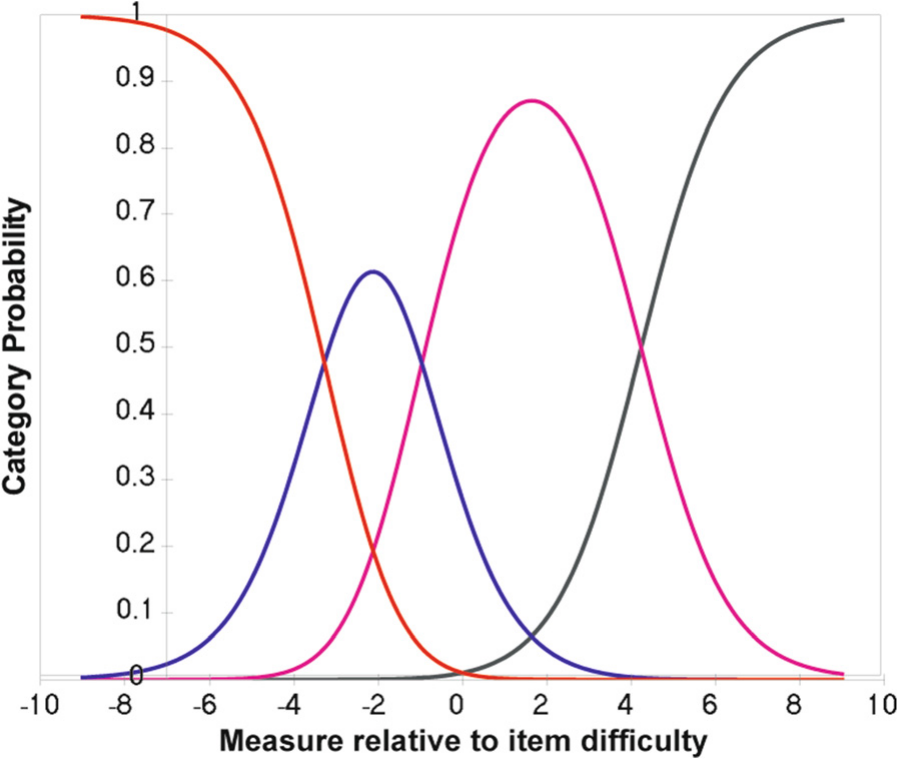
Second rating scale category probability curve. This figure displays the category probability curves for the second rating scale which corresponds to item 2 (Are you satisfied or dissatisfied with your present vision), demonstrating ordered thresholds

**Fig. 3:**
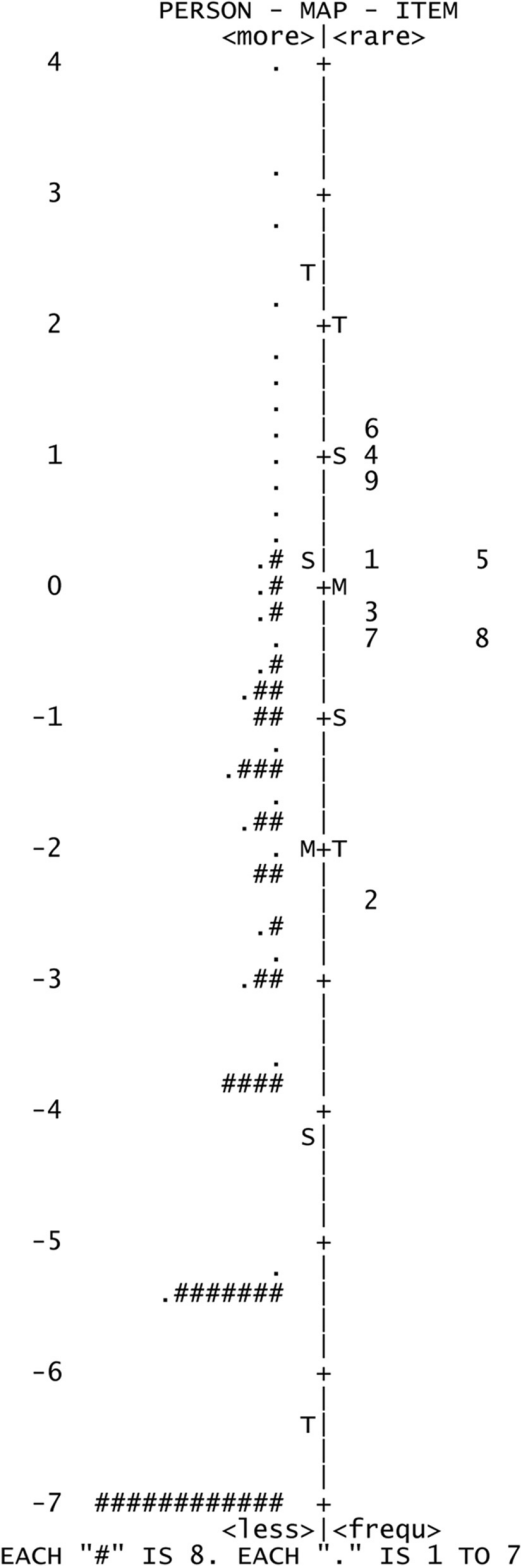
Person-item map, with persons on the left and items on the right. The mean difference in item difficulty and person ability is 2.04 logits

Response categories functioned as intended with ordered response options for the two rating scales as evidenced in the category probability curves depicted in Figs. 1 and 2. The questionnaire was found to be unidimensional with item fit statistics ranging from 0.73 to 1.34 (Table 2). Further principal components analysis testing revealed that the unexplained variance explained by the first, second, third, fourth and fifth contrasts were 1.8, 1.3, 1.2, 1.1 and 0.8 eigenvalue units respectively. As all contrasts were less than 2.0 eigenvalue units, this further evidences unidimensionality. There was adequate person separation with a ratio of 2.04, which is greater than the 2.0 cutoff. There was no significant differential item functioning between the different patient groups with values ranging between −0.46 and 0.47 logits. There was significant mistargeting as shown in person-item map in Fig. 3 and the mean value between the item and patient scores was 2.04 logits. Considering pre-operative data only, the targeting improves to 1.52. Further considering pre-operative first eye surgery only, the targeting improves to 1.33.

**Table 2:** Infit and outfit statistics for the nine items of the Catquest-9SF questionnaire

Item description	Infit	Outfit
	MNSQ	MNSQ
1. Do you experience that your present vision is giving you difficulty in any way in your everyday life?	1.04	0.91
2. Are you satisfied or dissatisfied with your present vision?	1.31	1.34
Do you have difficulty with the following activities because of your vision? If so, how much?		
3. Reading text in the daily paper	0.87	0.73
4. Recognise the faces of people you come across	1.08	0.94
5. See prices when shopping	0.84	0.81
6. Seeing to walk on uneven ground	1.05	1.00
7. See to do handwork, woodwork, etc.	0.95	1.11
8. Reading text on TV	0.86	0.80
9. See to carry on an activity/hobby you are interested in	0.86	0.79

*Abbreviation: MNSQ =* mean square

### Catquest-9SF outcomes

Data were assessed for normality using the Shapiro-Wilk test and were found to be non-normally distributed. Hence, the median, 25^th^ percentile and 75^th^ percentile are reported. Non-parametric Friedman tests were used to assess the change in visual disability scores with the Catquest-9SF pre-operative to post-operatively. Catquest-9SF scores are derived via Rasch analysis and more negative scores (or less positive) indicate better visual disability. Table 3 shows the change in Catquest-9SF scores pre- and post-operatively for the four groups of patients: first and second eye surgeries, with and without ocular comorbidity. There was an improvement in all four groups of patients and the results are shown in Table 4. A Kruskal-Wallis H test was performed to determine if the improvement in visual disability following cataract surgery between the four groups was statistically significant. The distributions of the data for the four groups were similar by visual inspection of a boxplot that is a requirement of the Kruskal-Wallis H test. The median improvement in visual disability was not statistically significant between the four groups, *X*^*2*^*(3)* = 5.445, *p* = 0.142. As there were no statistically significant differences, post hoc tests were not performed.

**Table 3:** Median, 25^th^ percentile, 75^th^ percentile, Chi-squared and *P*-value for first and second eye (with and without ocular comorbidity) pre- and post-operative Catquest-9SF scores (logit scale)

	First eye		Second eye	
	With comorbidity	Without comorbidity	With comorbidity	Without comorbidity
Pre-operative Catquest-9SF score				
Median	−1.40	−1.22	−1.55	−1.55
25^th^ percentile	−2.24	−2.28	−3.94	−3.23
75^th^ percentile	−0.11	−0.11	−0.11	−0.63
Post-operative Catquest-9SF score				
Median	−5.12	−5.12	−5.12	−5.12
25^th^ percentile	−6.78	−6.78	−6.78	−6.78
75^th^ percentile	−1.64	−3.76	−1.55	−3.63
*χ* ^2^ Chi-square	15.39	59.28	14.73	45.46
*P*-value	<0.001	<0.001	<0.001	<0.001

**Table 4:** Median improvement (logit scale) in Catquest-9SF scores for the four groups of patients

	First eye		Second eye	
	With comorbidity	Without comorbidity	With comorbidity	Without comorbidity
Median improvement in Catquest-9SF score	1.92	3.57	1.44	2.94

### Correlations

Spearman rank correlation coefficients were calculated to assess the correlation between pre-operative Catquest-9SF scores and LOCS III cataract grading. There was no correlation between Catquest-9SF scores and nuclear opalescence (*r*_s_ = 0.049, *p* = 0.478), nuclear colour (*r*_s_ = 0.008, *p* = 0.909), cortical (*r*_s_ = 0.066, *p* = 0.341), and posterior subcapsular (*r*_s_ = 0.048, *p* = 0.494). Filtering patients into individual cataract morphologies, there were 24 patients with nuclear only cataracts, 3 with cortical only cataracts and 25 with posterior subcapsular only cataracts. Due to the small sample size in the cortical only cataract group, a correlation coefficient was not calculated. For the nuclear only cataract group there was no statistically significant correlation with the Catquest-9SF score (*r*_s_ = 0.244, *p* = 0.250) and similarly for the posterior subcapsular cataract group there was no statistically significant correlation (*r*_s_ = 0.003, *p* = 0.987).

## Discussion

The Catquest-9SF was successfully translated into Italian and subsequent psychometric assessment with Rasch analysis indicated it was unidimensional in terms of adequate fit statistics and a principal components analysis. The response categories were ordered and functioned as intended. It was found to be precise with a person separation ratio of 2.04 logits and it was free from differential item functioning.

There was significant mistargeting with a mean difference in item difficulty and person ability of 2.04 logits. In an ideal situation there should be no difference between item difficulty and person ability. Targeting issues are usually due to floor or ceiling effects, which is the probable cause for the mistargeting in this study as there were large numbers of patients with no difficulties and high levels of satisfaction following surgery. This finding is in line with previous studies with this questionnaire, which similarly combined pre- and post-operative data and assessed targeting. A Swedish study involving 10,886 patients found a mean difference in item difficulty and person ability of 1.21 logits [11]. A further Swedish study involving 846 patients found a mean difference of 1.95 logits [27]. A two centre study based in Germany and Austria involving 210 patients found a mean difference of 1.69 logits [28]. An Australian and Chinese study involving pre-operative patients only found better person to item targeting, presumably due to higher levels of visual disability and less floor (or ceiling) effects depending on response option polarity [29, 30]. Hence, the mistargeting found in this study is indicative of the low levels of post-operative visual disability and high satisfaction experienced in this sample of patients. In the present study, targeting improved to 1.52 when pre-operative data was used only (first and second eye surgery), and improved further to 1.33 when only pre-operative first eye surgery data was used. Additionally, it may suggest that this cohort of Italian patients had less pre-operative visual disability compared to the other populations. Similar to previous studies in the literature, the most difficult item was ‘Are you satisfied or dissatisfied with your present vision’. The easiest item was ‘Seeing to walk on uneven ground’ for the Italian patients.

Focusing on the patient reported outcomes of the cohort of patients in this study, patients undergoing second eye cataract surgery had less visual disability than those undergoing first eye surgery, however all four groups had statistically significant improvements in visual disability following surgery with all four groups having a median post-operative Catquest-9SF score of −5.12. Despite larger median improvements in visual disability in the groups without ocular comorbidity, there was no statistically significant difference in the median improvement among the four groups. This finding adds to the mounting evidence of the beneficial gains with second eye surgery that helps support the argument against restrictions on second eye surgery in some countries [31–33].

The pre-operative Catquest-9SF score was compared to LOCS III cataract grading to assess if there was a correlation between the variables. This comparison is not without limitations, for example, the LOCS III is composed of grading for nuclear, cortical and posterior subcapsular cataracts and invariably many patients will have mixed cataracts. Other confounding factors with the analysis include the effect of ocular comorbidities, other eye status, and ocular dominance. Additionally, patients frequently are not wearing their most recent refractive correction due to the fact this will change post-operatively, which may influence the pre-operative visual disability. Further, posterior subcapsular cataracts are known to cause significant visual symptoms even in mild forms. Attempting to try to limit some of these limitations, patients were filtered into their respective cataract morphologies of nuclear only, cortical only and posterior subcapsular only cataracts. However, this had the knock on effect of a significantly reduced sample size. Nevertheless, the analysis indicated no significant correlation between the variables. All that can be drawn from the comparisons is that there was no correlation found between the Catquest-9SF score and LOCS III cataract grading in this cohort of patients. This finding perhaps explains common clinical scenarios whereby cataract severity is out of proportion to the symptoms experienced and vice versa.

Detailed information with regards to the pre- and post-operative (where applicable) UDVA, SE refraction, CDVA, QoV scores, LOCS III cataract grading including comparisons between the four patient groups (first and second eye surgery, with and without ocular comorbidities) are available in a previous publication involving the same cohort of patients. In brief, improvements in QoV scores were found in all 4 groups [26]. There were no statistically significant differences among the 4 groups in the improvement in QoV scores or in the preoperative or postoperative scores. The symptom “Blurred vision” from the QoV questionnaire was correlated with posterior subcapsular cataract (*r*_*s*_ = 0.420, *P* = 0.04) [26].

## Conclusions

The Catquest-9SF was successfully translated into Italian and demonstrated robust psychometric properties. It is therefore suitable for use in Italian speaking patients with cataract and following cataract surgery. It is a concise questionnaire and should be considered for use in the routine clinical care of patients and not restricted to research studies. Significant improvements in visual disability were found in this cohort of Italian patients, including those having had first and second eye surgery with and without ocular comorbidity. There was no statistically significant difference in the improvements among the four groups. Finally, there was no correlation found between the pre-operative Catquest-9SF score and LOCS III grading.
